# Diabetes Mellitus, Extreme Insulin Resistance, and Hypothalamic-Pituitary Langerhans Cells Histiocytosis

**DOI:** 10.1155/2019/2719364

**Published:** 2019-06-23

**Authors:** Mathilde Sollier, Marine Halbron, Jean Donadieu, Ahmed Idbaih, Fleur Cohen Aubart, Corinne Vigouroux, Martine Auclair, Olivier Bourron, Marie Bastin, Géraldine Béra, Philippe Touraine, Jacques Young, Héléna Mosbah, Agnès Hartemann, Fabrizio Andreelli, Chloé Amouyal

**Affiliations:** ^1^Diabetology-Metabolism Dpt, Sorbonne Université, APHP, Institut Hospitalo–Universitaire de Cardiometabolisme et Nutrition (ICAN), Pitié-Salpêtrière-Charles Foix Hospital, 75013 Paris, France; ^2^Hemato-Oncology Dpt, Trousseau Hospital, APHP, 75012 Paris, France; ^3^Sorbonne Université, Inserm, CNRS, UMRS 1127, Institut du Cerveau et de la Moelle épinière, ICM, AP-HP, Hôpitaux Universitaires La Pitié Salpêtrière -Charles Foix, Service de Neurologie 2, Mazarin, 75013 Paris, France; ^4^Department of Internal Medicine 2, Sorbonne Université, APHP, Institut Hospitalo–Universitaire de Cardiometabolisme et Nutrition (ICAN), Pitié-Salpêtrière-Charles Foix Hospital, 75013 Paris, France; ^5^Inserm UMRS 938, Centre de Recherche Saint–Antoine, Sorbonne Université, Reference Center for Rare Diseases of Insulin Secretion and Insulin Sensitivity, Institut Hospitalo–Universitaire de Cardiometabolisme et Nutrition (ICAN), Paris, APHP, Saint Antoine Hospital, Paris, France; ^6^Nuclear Medicine Dpt, Sorbonne Université, APHP, Pitié-Salpêtrière-Charles Foix Hospital, 75013 Paris, France; ^7^Department of Endocrinology and Reproductive Medicine, Pitié-Salpêtrière Hospital, APHP, Reference Center for Rare Endocrine Diseases of Growth, Reference Center for Rare Gynecological Pathologies, Institut Hospitalo–Universitaire de Cardiometabolisme et Nutrition (ICAN), Sorbonne University, 75013 Paris, France; ^8^Pitié-Salpêtrière-Charles Foix Hospital, 75013 Paris, France; ^9^Reproductive Endocrinology Department, Paris Sud University, APHP, Le Kremlin-Bicêtre, 94275, France

## Abstract

**Background:**

Langerhans Cell Histiocytosis (LCH) is a rare inflammatory neoplasm characterized by an infiltration of organs by Langerin + (CD207+) and CD1a+ histiocytes. Diabetes insipidus is a frequent manifestation of the disease, while diabetes mellitus is very rare. We report the first case of a 20-year-old man suffering from hypothalamopituitary histiocytosis and diabetes mellitus with serum anti-insulin receptor antibodies.

**Case Presentation:**

A 20-year-old patient was admitted for the evaluation of growth delay and hyperphagia. HbA1c level and fasting blood glucose were in the normal range. The diagnosis of hypothalamopituitary histiocytosis was based on histological features after biopsy of a large suprachiasmatic lesion identified on magnetic resonance imaging (MRI). Association of vinblastine and purinethol was started followed by a second-line therapy by cladribine. During the follow-up, the patient was admitted for recurrence of hyperglycemic states and extreme insulin resistance. The screening for serum anti-insulin receptor antibodies was positive. Each episode of hyperglycemia appeared to be correlated with tumoral activity and increase in serum anti-insulin receptor antibodies and appeared to be improved when the disease was controlled by chemotherapy.

**Conclusion:**

We report the first description of a hypothalamopituitary histiocytosis associated with serum anti-insulin receptor antibodies, extreme insulin resistance, and diabetes. Parallel evolution of glucose levels and serum anti-insulin receptor antibodies seemed to be the consequence of immune suppressive properties of cladribine.

## 1. Background

Langerhans Cell Histiocytosis (LCH) is a rare disease characterized by an infiltration of organs by Langerin+ (CD207+) and CD1a+ histiocytes [1, 2]. Although it can occur at any time of life with an incidence of 4.6 cases per million habitants, LCH is more frequent in children (with onset at a peak age of 1-3 years) [3]. The pathogenesis of LCH is not clear but is likely linked to an inflammatory neoplasm. The definitive diagnosis relies on histology with expression of CD1a antigen or CD207 antigen according to the revised criteria of the Histiocyte Society [1]. Multiple tissues can be concerned by LCH but the most frequent are bones, skin, and pituitary [4]. Indeed, a recent review of endocrine manifestations showed that pituitary deficiency associated with diabetes insipidus (DI) is one of the most frequent endocrine diseases observed at onset of LCH [5]. Endocrine pancreas is rarely concerned and to our knowledge only five cases of pancreatic infiltration by LCH cells have been reported in the literature [6–10]. In addition, association between LCH and diabetes mellitus with or without hypothalamopituitary involvement has been published [11–15]. We report for the first time the case of a 20-year-old man presenting a hypothalamopituitary histiocytosis and positive for serum anti-insulin receptor antibodies with a follow-up characterized by alternating periods of diabetes mellitus with extreme insulin resistance and periods of normoglycemia.

## 2. Case Presentation

**Figure 1:**
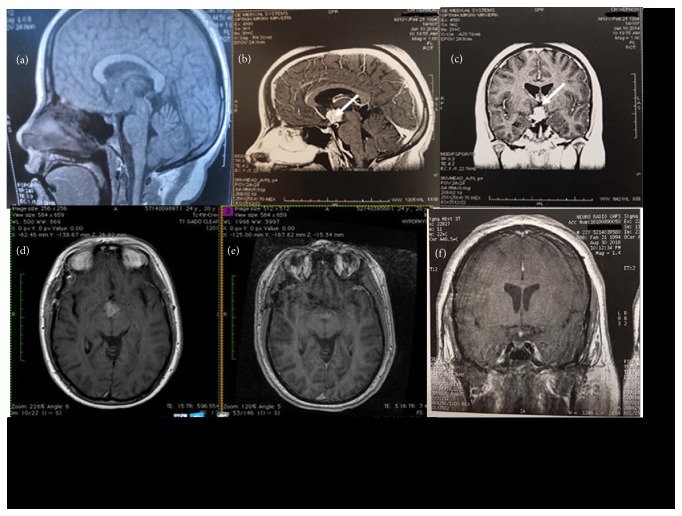
*Evolution of brain MRI during follow-up.* At diagnosis, the normal T1-weighted hyperintense signal of the posterior pituitary has disappeared (a). Contrast-enhanced hypothalamic mass after gadolinium infusion ((b) and (c)); compression of third ventricle and thickening of pituitary stalk (b). Comparison of brain MRI (T1 with gadolinium) at diagnosis (M0) (d) and at the end of the first-line therapy (e). MRI showed a significant decrease of the hypothalamic mass but the persistence of contrast enhancement after gadolinium. Brain MRI (T1 after gadolinium infusion) at M30 showing a hypothalamic lesion stable in size and with decreased hypersignal compared to previous MRI (f).

The patient #1509232 is a young male with a history of polyuria and polydipsia since he was 11 years old. This symptom was neglected. The patient had a normal academic progression and medical work-up (at age of 14 years and 17 years) excluding several times the diagnosis of diabetes mellitus, as no hyperglycemia has been detected. At the age of 20 years and 5 months he became polyphagic and his weight increased from 60 to 90 kg without any other complaint. Finally, 5 months later, a brain MRI was performed and a large hypothalamus mass (20x20mm) with thickening of the pituitary stalk and compression of the third ventricle was observed. A strong contrast enhancement was observed after administration of gadolinium (Figures 1(a), 1(b), and 1(c)).

The patient was admitted in the department of endocrinology and the biological explorations concluded to a panhypopituitarism associated with diabetes insipidus. At the initial assessment, the following was also discovered: (i) the patient presented stage I obesity (BMI=26.6 kg/m2), (ii) delayed bone age relative to chronological age (resp., 16 years for a chronological age of 20), and (iii) a growth delay with a deceleration of linear growth at age of 15 years.

A stereotactic biopsy of the brain mass was performed at age of 21 years (Table 1, M1) and immunohistochemistry showed positive staining for CD1a and PS100, supporting the diagnosis of LCH. Presence of BRAF V600E mutation was explored because this mutation in LCH is associated with more severe disease than did those with wild-type BRAF and irreversible damage, such as neurologic and pituitary injuries [16]. In addition, presence of BRAF V600E mutation may offer the possibility of a targeted therapy by BRAF inhibitor (vemurafenib or PLX8394, a second-generation BRAF inhibitor) [17]. Unfortunately, the mutation was not observed and this has limited the possibility to use BRAF inhibitors). Disease work-up failed to find any extra pituitary-hypothalamic extension.

The patient was referred to the oncology department to start vinblastine (one infusion of 10mg per week) and purinethol (100mg per day) according to the French guidelines HL2010 protocol [18]. He did not receive corticosteroids. The patient signed an informed consent to be enrolled in the French LCH registry according to regulation [19].

One month after, the patient was admitted to the emergency room for altered consciousness (Table 1, M0). The blood tests showed hypernatremia at 164mmol/l and hyperglycemia at 25.8mmol/l without ketosis. Importantly, blood glucose levels were in the normal range 6 months before and at the time of the brain biopsy. After continuous administration of fluids and rapid acting insulin analogue (124UI/24hr), he was admitted to the diabetology department.

The search for type 1 diabetes (anti-GAD and anti-IA2 antibodies) was negative. C-peptide level was 9*µ*g/l in the fasting state (normal range: 0.8-4.2 *µ*g/l). The lipid profile showed hypertriglyceridemia at 5.7mmol/l (5,4g/l) and low HDL-cholesterol level at 0.41 mmol/l (0.16g/l). Blood pressure was normal at 125/68mmHg. The abdominal CT-scan found liver steatosis without any pancreatic abnormalities. The weight gain of 19kg during the last 3 months (body mass index: 32kg/m^2^) was linked to eating disorder (especially binge-eating disorder). It was decided to continue the first-line therapy up to 6 infusions of vinblastine.

Intriguingly, soon after the diagnosis of diabetes mellitus, daily insulin needs grew up to 400UI per day (4.2UI/kg), indicating a significant insulin resistance state. We did not observe acanthosis nigricans or loss of subcutaneous tissue and we excluded hypersecretion of hyperglycemic hormones. We tested the hypothesis of antibodies against insulin receptors as a cause of insulin resistance. The screening for serum anti-insulin receptor antibodies was performed using a radioreceptor assay as described [20, 21]. In summary, Chinese Hamster Ovary cells overexpressing insulin receptor (CHO-IR) were incubated or not for 90 min at 22°C with control and patient's serum at different dilutions, washed, and then incubated for 90 min at 22°C with a tracer concentration of ^125^I-insulin with or without unlabeled insulin.

The ability of patient's serum to inhibit binding of [^125^I]insulin was expressed as a percent decrease of insulin binding at 1:3 dilution as compared to the reference value. At the diabetes diagnosis, insulin receptor antibodies were positive (42% decrease in insulin binding) (Table 1), allowing the diagnosis of type B insulin resistance, a disease often associated with autoimmune disorders or hematological diseases. We then excluded systemic lupus erythematous, multiple myeloma, and Hodgkin's disease. Interestingly, insulin receptor antibodies levels decreased when first-line therapy was achieved (19% decrease in insulin binding) in parallel to a spectacular decrease in daily insulin needs (Table 1). Insulin therapy could be discontinued and replaced by a monotherapy with metformin (850mg twice a day). HbA1c remained stable at 6.5% (48 mmol/mol) until M8. In contrast, no improvement of binge-eating disorder was observed and body mass index kept increasing significantly during this period (Table 1).

Seven months after initial diagnosis of diabetes mellitus, intensification of hypoglycemic therapy with sulfonylurea and liraglutide LAR added to metformin was needed because glucose levels increased. The change in glucose levels was observed at the same time as a further increase of serum insulin receptor autoantibodies at M8 and M10 (43 and 42 percent decrease of insulin binding to its receptor upon incubation of CHO-IR cells with patient's serum at 1:3 dilution, resp.) (Table 1). Although a significant decrease of the size of the brain mass was observed when compared to initial evaluation, persistence of contrast enhancement after gadolinium administration suggested an incomplete response to therapy (Figures 1(d) and 1(e)).

A second-line therapy was started, consisting in 6 cycles of cladribine (5mg/m2/24h, 5 days/week, each month) from month 12 to month 18 after the diagnosis. At M30, brain MRI showed a decrease of both tumor volume and contrast enhancement (Figure 1(f)). In addition, glucose levels were controlled with the association of metformin and liraglutide (HbA1c level decreased to 7.5% or 58mmol/mol). Diabetes improvement was associated with a decrease of insulin receptor antibodies levels until M31. Unfortunately, a new recurrence of uncontrolled diabetes (HbA1c: 11.2%, 99 mmol/mol) occurred at M32 needing insulin therapy. Insulin receptor antibodies were once again positive (36% inhibition of insulin binding by patient's serum at 1:3 dilution), while brain MRI did not reveal tumor recurrence.

**Figure 2:**
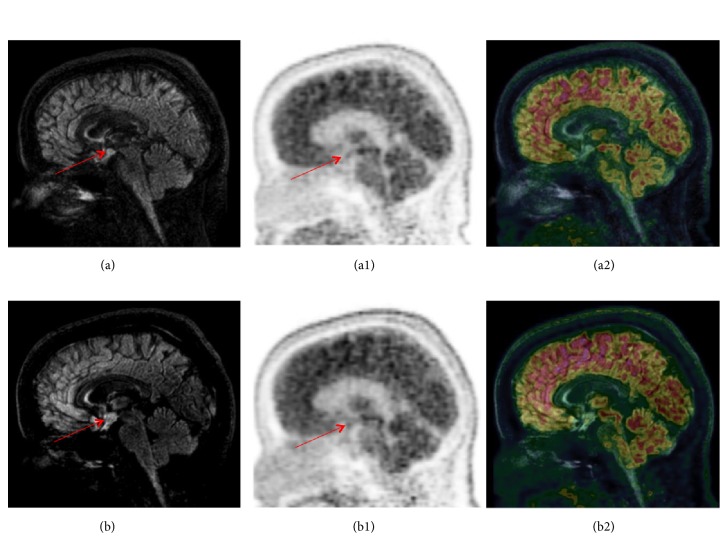
*Sagittal PET-MRI brain imaging.* Sagittal PET-MRI brain imaging with [18F]FDG at M32 (a, a1, a2) and at M35 (b, b1, b2) of follow-up. Nodular thickening of the floor of the third ventricle and pituitary infundibulum, in hyperintense FLAIR, without [18F]FDG-uptake, measuring 7 mm in height x 13 mm in the axial plane, without significant enhancement, globally unchanged from the previous MRI. Linear and fine enhancement of the pituitary infundibulum with stable appearance.

**Figure 3:**
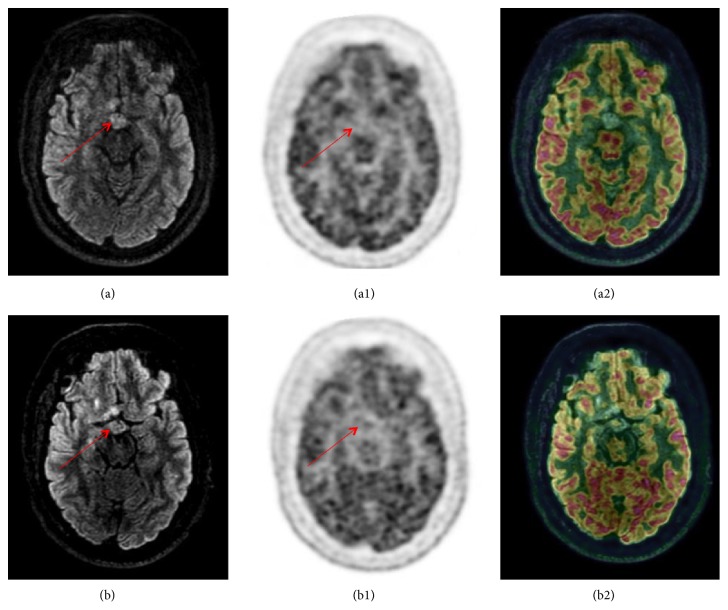
*Axial PET-MRI brain imaging.* Axial PET-MRI brain imaging with [18F]FDG at M32 (a, a1, a2) and at M35 (b, b1, b2) of follow-up.

It was decided to initiate new cycles of cladribine. The patient received a first cycle at M32 after diagnosis and a second one month later. During this period, insulin daily doses decreased from 300UI to 36UI at the end of cladribine cycles. Additionally, HbA1c level decreased from 11.2% to 8.6% (from 99 to 70 mmol/mol). The PET-MRI made during the relapse of diabetes at M32 showed a nodular thickening of the floor of the third ventricle and pituitary infundibulum with no [18F]-FDG-uptake (Figures 2 and 3). Control of diabetes was maintained until M35 and PET-MRI was unchanged at that time (Figures 2 and 3). The follow-up was marked by a very complex clinical disorder, with behavioural difficulties, polyphagy and agressivity; neurological difficulties, loss of memory; and very instable basal metabolic situation, hyperglycemia, and recurrent hypernatremia episodes. Four years after the diagnosis of LCH, the patients is actually frequently hospitalized in a psychiatric hospital.

## 3. Case Discussion

We reported a case of isolated hypothalamic-pituitary LCH and panhypopituitarism, with multiple episodes of extreme insulin resistance and diabetes mellitus. Each episode appeared to be associated with tumoral activity and appeared to be improved when brain tumor was controlled by chemotherapy.

Each period of hyperglycemia was characterized by important specificities. First, blood glucose levels were extremely high at each recurrence of diabetes and induced massive dehydration and hypernatremia in a context of diabetes insipidus. A second major characteristic was the insulin resistance state. Important doses of insulin (4UI/kg/D) were necessary to maintain glycemia around 11 mmol/l, regardless of how insulin was administered, intravenously or subcutaneously. The hyperglycemia periods alternated with periods of improvement of glucose homeostasis; oral hypoglycemic monotherapy with metformin was then sufficient to achieve the target of HbA1c (< 6.5% or 48 mmol/mol). Such fluctuations in glucose homeostasis and insulin resistance raise the question of the etiology of this diabetes. Low levels of ketone bodies and negativity of islet antibodies contributed to excluding the diagnosis of type 1 diabetes. The most frequent etiologies of insulin resistance (such as infectious disease, lipodystrophy, excessive secretion of hyperglycemic hormone, or corticosteroids therapy) were absent. In addition, the increase in body weight observed before onset of diabetes was not sufficient to explain by itself such an important level of insulin resistance at the discovery of diabetes.

Thus, we hypothesized that insulin resistance could be triggered by serum anti-insulin receptor antibodies. Indeed, in this condition (known as type B insulin resistance), such antibodies are known to reduce insulin (exogenous or endogenous) transduction signaling in insulin target tissues [22]. Arioglu et al. compared 24 patients with type B insulin resistance and admitted in their clinical center with representative case reports gathered from the literature [23]. Most type B insulin resistance was described in patients with systemic autoimmune diseases (especially systemic lupus erythematous) or associated with multiple myeloma and with Hodgkin's disease. All of these diseases were absent in our patient.

In our case, the positivity of anti-insulin receptor antibodies in blood and the observation of resistant hyperglycemic state despite intravenous or subcutaneous insulin therapy strongly suggested a possible causal link between these antibodies and the pathophysiology of diabetes. Accordingly, during the follow-up, we established a parallel evolution of serum insulin receptor antibodies and glucose homeostasis. In contrast, no correlation was found between serum insulin receptor antibodies (or control of diabetes) and body weight.

Whether positivity of insulin receptor antibodies and LCH is a direct relationship or a fortuitous association is not clear. Indeed, cases of diabetes mellitus associated with LCH are rare if we except a transient glycemic intolerance during corticoid steroid therapy. Among the 3200 cases in the national French LCH registry, which enrolled 56 cases of hypothalamic tumor, we failed to find any similar conditions [19, 24, 25]. In the literature, only nine cases with diabetes and LCH have been so far reported and summarized in Table 2.

As in our case, seven of these five observations have in common a diabetes insipidus as first symptom of LCH in the hypothalamic-pituitary region [7, 11–13, 15]. The onset of diabetes mellitus was described as a hyperglycemic hyperosmolar syndrome in three previously published observations [11, 12, 14]. As for our patient, hyperosmolarity was probably the consequence of massive glycosuria and polyuria, which leads to massive dehydration in a context of diabetes insipidus. In few observations, onset of diabetes was observed after a body weight gain in relation with hyperphagia when histiocytosis developed in the infundibulum as in our case [7, 11, 13, 15]. Only one publication reported massive insulin needs as observed in our case [12]. Lastly, localization of LCH in pancreas has been exceptionally observed in two cases without diabetes. Yu RC et al. reported the postmortem findings in an 18-month-old boy with infiltration of pancreas by cells with the characteristic features of LCH [9]. However, the islets of Langerhans were preserved. Muwakkit et al. published a case of disseminated LCH in a 4-week-old infant with a cystic lesion in the pancreas discovered at abdominal CT [10]. This lesion improved with systemic therapy of LCH.

The first-line treatment used for LCH remains empirically derived chemotherapy [18], whereas cladribine has been proposed as a second-line therapy [26]. In contrast with the other cases published, we showed that therapy of LCH had beneficial effect on diabetes mellitus. The patient first received vinblastine and then cladribine as a second-line treatment because vinblastine cycles had only partial effect on brain mass volume. Cladribine therapy provided an excellent effect on brain mass volume. Improvement of glucose homeostasis (in parallel to decline of anti-insulin receptor antibodies in blood) was observed during first- and second-line therapy of LCH. Thus, it could be postulated that reduction of the brain mass volume and/or LCH activity will explain almost in part the improvement of glucose homeostasis. Nevertheless, it is difficult to determine if metabolic amelioration was a consequence of tumor shrinkage or an immune-suppressive effect but some arguments seemed to favor the latter. Indeed, recurrence of hyperglycemia exacerbation at M32 was not associated with changes of volume of the brain mass or its metabolic activity assessed by PET-MRI brain imaging with [18F]FDG. In contrast, this episode was closely correlated with the level of antibodies. Furthermore, at our knowledge, there is no publication about hypothalamic syndrome and anti-insulin receptor antibodies.

## 4. Conclusion

For the first time, we describe a case of LCH and diabetes and anti-insulin receptor antibodies. In this unique case, insulin resistance could be explained by presence of serum anti-insulin receptor antibodies with a direct link between anti-insulin receptor antibodies and LCH. We hypothesized that recurrent insulin resistant states can be assimilated to a paraneoplastic syndrome. We observed a positive effect of vinblastine and cladribine on glycemic control, probably explained by immune control independent of tumor size evolution.

## Figures and Tables

**Table 1:** *Evolution of blood glucose levels, BMI, and anti-insulin receptor antibodies in blood*. Evolution of fasting blood venous glycemia (measured at each administration of chemotherapy or at each assessment of anti-insulin receptor antibodies) and the ability of patient's serum to inhibit insulin binding to its receptor. The reduction of [^125^I]insulin binding in the presence of patient's serum (used at 1:3 dilution) was measured on CHO cells overexpressing insulin receptor as described (18-20). Ins: insulin. Met: metformin.

	IMC (kg/m2)	Anti-insulin receptor antibodies	Blood glucose levels (mM)	Admissions	Therapy for diabetes	Therapy for histiocytosis
M-1	24		4,4	Stereotactic biopsy		
M0	31,5		25,8	Hyperosmolar hyperglycemic coma	Ins	Vinblastine + purinethol
M1	32		15		Ins + Met + sulfamides	Vinblastine + purinethol
M2	31	42	13		Ins + Met + sulfamides	Vinblastine + purinethol
M2	32,1	35	8,2		Ins + Met + sulfamides	Vinblastine + purinethol
M2	32,1	19	5,5		Met	Vinblastine + purinethol
M8	33,8	43	13,7	Relapse of hyperglycemia	Met + sulfamides + GLP-1 analog	Vinblastine + purinethol
M10	35,2	42	11		Met + sulfamides + GLP-1 analog	Cladribine
M20	35,2	49	11		Met + sulfamides + GLP-1 analog	Cladribine
M28	37,6	30	14,3		Met + sulfamides + GLP-1 analog	
M31	37,6	2	5,5		Met + GLP-1 analog	
M32	39	36	22	Relapse of hyperglycemia	Ins	Cladribine
M33	39	32	17		Ins	Cladribine

**Table 2:** Characteristics and specific organ involvement of cases of diabetes mellitus associated with LCH.

Age at diagnosis of histiocytosis (years)	Sex	Diagnosis of histiocytosis	Diabetes insipidus	Delay between diagnosis of histiocytosis and diabetes	Pathophysiology of diabetes	Daily insulin needs	Reference
31	M	Bone biopsy	+	2 years	Central obesity, insulin resistance	NA	[11]

60	F	Autopsy (histiocytes cells in vertebral marrow, pituitary, lung	+	3 years	Central obesity, insulin resistance	3300 UI/day	[12]

26	M	Autopsy (histiocytes cells in pancreas)	+	2 years	Destruction of pancreatic islets by pancreas infiltration of histiocytosis	80UI/day	[7]

28	F	Lymph node biopsy	+	Few months	Central obesity, insulin resistance	NA	[13]

NA	M	Autopsy (histiocytes cells in skin, liver, bones)	-	6 months	NA	NA	[14]

54		Central nervous system, bones	+	NA	Glucocorticoids, overweight	NA	[15]

60	F	Central nervous system, bones	+	NA	Overweight	NA	[15]

54	M	Gengives	+	NA	Glucocorticoids, overweight	NA	[15]

45	F	Bones, lung, lymph nodes	-	NA	Obesity	NA	[15]
